# Synchrotron X-ray tomography sheds light on the phylogenetic affinities of the enigmatic thylacocephalans within Pancrustacea

**DOI:** 10.1098/rspb.2025.1612

**Published:** 2025-11-12

**Authors:** Thomas Laville, Marie-Béatrice Forel, Andrew King, Sylvain Charbonnier

**Affiliations:** ^1^ Fakultät für Biologie, Ludwig-Maximilians-Universität München, Planegg-Martinsried 82152, Germany; ^2^ Centre de Recherche en Paléontologie – Paris - UMR 7207, Muséum National d'Histoire Naturelle, Paris, Île-de-France 75005, France; ^3^ PSICHÉ Beamline, Synchrotron SOLEIL, Gif-sur-Yvette 91190, France

**Keywords:** Euarthropoda, Jurassic, La Voulte Lagerstätte, Pancrustacea, synchrotron X-ray tomography, tagmatization, appendages

## Abstract

Thylacocephala is one of the most puzzling groups of fossil marine euarthropods. They are known from the Silurian to the Cretaceous and are characterized by a univalve shield, hypertrophied compound eyes, three pairs of large, raptorial appendages and an 8 to 22 segmented posterior trunk. Despite this knowledge of their anatomy, numerous questions remain on their phylogenetic affinities. Usually considered as pancrustaceans, they have been tentatively placed within various pancrustacean ingroups such as thecostracans, malacostracans or remipeds. This uncertainty on their phylogenetic relationships is mostly due to a lack of knowledge on their body organization, especially on the number, nature and morphology of their cephalic, raptorial and posterior trunk appendages. We applied synchrotron X-ray microtomography to exceptionally preserved specimens of *Dollocaris ingens* from the Middle Jurassic La Voulte-sur-Rhône Lagerstätte, Ardèche, France (Callovian, approximately 165 Ma). It revealed unique details of their tagmatization. For the first time, we demonstrate the unambiguous presence of several cephalic appendages (i.e. mandibles, maxillules, maxillae), an anterior trunk bearing the raptorial appendages and a posterior trunk. These new anatomical data allowed us to test the phylogenetic affinities of thylacocephalans among arthropods. The phylogenetic analyses strongly suggest that thylacocephalans form a pancrustacean monophyletic group, being closely related to malacostracans.

## 1. Introduction

Pancrustacea is a highly diverse group of euarthropods, with an extensive fossil record dating back to the Cambrian [1]. Among presumed fossil representatives are the enigmatic thylacocephalans. Known through the Palaeozoic and the Mesozoic, thylacocephalans are puzzling marine euarthropods characterized by key anatomical features such as a shield enveloping most of the body, a pair of compound eyes sometimes hypertrophied, three pairs of raptorial appendages, eight pairs of gills and an 8 to 22 segmented posterior trunk bearing appendages [2]. They are generally considered to be pancrustaceans based on the presence of an antennula and an antenna [3], of mandibles [4], of several proximal endites on the basis of their appendages [2], of dorsal sensory organs [5] and on the similarity of their organ systems with those of other pancrustaceans [6]. However, their position relative to other pancrustacean ingroups is still unclear. They have been tentatively grouped together with thecostracans [7], malacostracans [6,8,9] and more recently with remipedians [2]. So far, the affinities of thylacocephalans have only been tested through two phylogenetic analyses, linking them either with malacostracans [10] or with mandibulates [11]. However, these results should be treated with caution as numerous details of their anatomy, in particular the internal anatomy, were unknown at the time of the first study. Additionally, the various hypotheses regarding their tagmatization have not been tested. Despite extensive knowledge of their anatomy, their tagmatization is highly controversial as the exact number, nature and morphology of their cephalic, raptorial and posterior trunk appendages are still poorly known.

This lack of knowledge on their appendages is mainly due to the fact that most thylacocephalan species are only known by their shield. Material from exceptionally well-preserved deposits, also known as Konservat-Lagerstätten, could help tackle this issue, as they can provide a unique insight into the external and internal anatomy of thylacocephalans. Among them is the 165 Ma Konservat-Lagerstätte of La Voulte-sur-Rhône (Callovian, Ardèche, France). It is renowned worldwide for its diverse euarthropod fauna [12], including six species of thylacocephalans [13], as well as for the high degree of morphological preservation of its fossils, including fully articulated echinoderms [14] but also cephalopods [15], erymid lobsters [16] and thylacocephalans preserved with soft parts [6]. Indeed, rare details of the internal anatomy of the thylacocephalan species *Dollocaris ingens* Van Straelen, 1923, including the digestive and the respiratory systems, have been recently uncovered thanks to the use of laboratory X-ray microtomography [6]. Cephalic, raptorial and trunk appendages were, however, not studied due to little X-ray contrast exhibited by the specimens.

To cope with this issue, phase-contrast synchrotron X-ray microtomography was performed, as previously done for La Voulte lobsters and cephalopods, disclosing unique three-dimensional soft tissue preservation [15,16]. The investigation of *D. ingens* by this technique revealed unique details of its body organization and allowed us to test the phylogenetic affinities of thylacocephalans.

## 2. Material and methods

### (a) Material

Six specimens of *D. ingens* deposited in the Palaeontological collections of the Muséum National d’Histoire Naturelle (Paris, France) were scanned for this study (MNHN.F.A29258, A29277, A97318–A97320, R50931). They come from the marls of the Middle Jurassic (early Callovian) La Voulte-sur-Rhône Lagerstätte in France [12]. They are preserved in three dimensions in carbonate nodules that were mechanically opened. In addition to the aforementioned specimens, polished cross-sections made by Sylvie Secretan were studied to characterize the structure of the shield (MNHN.F.R06054) as well as one compressed specimen to study the posterior trunk (MNHN.F.A29251).

### (b) Photography

Specimens were photographed with a NIKON D700 camera equipped with a NIKON AF-S NIKKOR 35 mm f/1.8 g ed macro lens. Photographs of MNHN.F.A29251 as well as details of the polished cross-sections were made using an Axio ZOOM.V16 stereoscopic microscope equipped with a DeltaPix Invenio-20EIII camera under cross-polarized light, at the Centre de Recherche en Paléontologie—Paris (CR2P).

### (c) Tomography

Phase-contrast synchrotron X-ray microtomography was performed at the PSICHÉ beamline (Pression Structure Imagerie par Contraste à Haute Énergie) of the SOLEIL synchrotron (Saint-Aubin, France). Each specimen was scanned with a voxel size of 11.139 or 22.278 µm (corresponding to 2 × 2 and 4 × 4 pixel binning, respectively) and a single propagation distance of 1150 mm. A pink beam of 120 keV was selected from the insertion device spectrum by filtering the beam with 19.6 mm of copper. The detector camera was a Hamamatsu ORCA Flash 4.0 sCMOS, and the scintillator a 500 µm LuAG:Ce, coupled with a pair of 100 mm Hasselblad objectives in a tandem configuration. Due to the limited field of view and the size of the samples, a series of acquisitions with vertical movement of the sample was recorded. We performed scans of 1500 projections of 0.017 s exposure time over 360° with phase retrieval using a Paganin process. The scans were then assembled in a mosaic in order to reconstruct the whole volume. Volumes were reconstructed using filtered back projections with ESRF PyHST2 software [17]. The tomographic and three-dimensional rendering data are available on Zenodo (see Data accessibility section) [18,19].

### (d) Segmentation

Among the six scanned specimens, the two best-preserved were segmented (MNHN.F.A97320, R50931). Crop, rotation and conversion to 8 bit greyscale were achieved prior to the segmentation using ImageJ2 [20]. Segmentation was performed using Materialise Mimics innovation suite 24.0 (research edition). It was mostly achieved through manual segmentation of selected slices and interpolation between these slices. When possible, semi-automatic segmentation tools were used, in particular, thresholding associated with the *region grow* function to separate objects of similar attenuation. Three-dimensional rendering was made with Meshlab 2022.02 [21]. Cleaning (*removing isolated pieces* with a threshold of 300 voxels*, removing duplicated faces* functions and manual cleaning), reorientation (*reorienting all face coherently* function) and remeshing filters (*repairing non-manifold edges*, *closing holes* and *quadric edge collapse decimation* functions with preserve normal and topology checked) were applied.

### (e) Scanning electron microscopy

The shield ornamentation of specimen MNHN.F.R50931 was studied by scanning electron microscopy. The chemical contrast observations (back-scattered electrons) were made using a Hitachi SU3500 scanning electron microscope from the MNHN electron microscopy and microanalysis technical platform. The specimen was not coated with metal.

### (f) Phylogenetic analyses

Phylogenetic affinities of thylacocephalans were studied based on the morphological character matrix of [22], a modified version of the one of [23]. The matrix contained 276 characters and 111 taxa. Thirteen taxa were added, including two species of Thylacocephala (*D. ingens* and *Thylacares brandonensis* C. Haug *et al.*, 2014), reaching a total of 124 taxa. Character codings for some already present taxa were modified according to the most recent studies (see electronic supplementary material, table S1). Two Bayesian inference analyses were performed using MrBayes 3.2.7 [24]. Following [22], a first analysis was performed with backbone topological constraints, resulting from phylogenomic studies (analysis with constraints, AC). Some constraints were modified from the original analysis (see electronic supplementary material, document S1). The second analysis was performed without topological constraints (analysis without constraintsAW). Both analyses were performed under the Mkv model with a gamma-distributed rate variation across sites. Three independent runs of 2 × 10^8^ Markov chain Monte Carlo generations for each analysis were performed. For each analysis, four Markov chains (one cold, three heated) were used, the relative burn-in fraction was set to 20% and the chains were sampled every 1000 generations. The temperature parameter was set to 0.1. Tracer v.1.7.1 [25] was used to assess the convergence of each replicate. The graphical representation of the maximum clade credibility trees was achieved using FigTree v.1.4.4 software. The character list is available in electronic supplementary material, document S1, while the matrix and the scripts for the analyses are available on the Zenodo platform (see Data accessibility section) [26].

## 3. Results

### (a) Taphonomy

**Figure 1.**
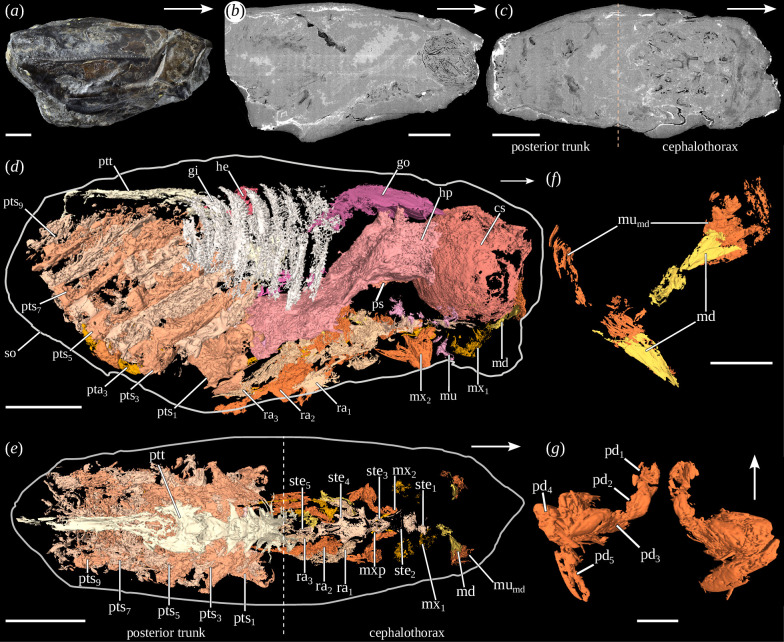
Anatomy (*a–f*) and cephalic appendages morphology (*g*, *i*) of *Dollocaris ingens* Van Straelen, 1923 (MNHN.F.R50931). (*a*) Right lateral view of the shield, (*b*) sagittal virtual section, (*c*) frontal virtual section, (*d*) lateral view without the shield (three-dimensional rendering), (*e*) dorsal view without the shield and organ systems (three-dimensional rendering), (*f*) posterior view of the mandible (three-dimensional rendering). The mandibles have been moved relative to each other for visualization purposes, (*g*) dorsal view of the maxillae. White arrows indicate the anterior side. cs, cardiac stomach; gi, gills; go, gonad; he, heart; hp, hepatopancreas; md, mandible; mu, muscle; mu_md_, mandibular muscles; mx_1_, maxillula; mx_2_, maxilla; mxp, maxilliped; pd_1–5_, podomeres; ps, pyloric stomach; pta_3_, posterior trunk appendage 3; pts_1–9_, posterior trunk segments; ptt, posterior trunk tergal part; ra_1–3_, raptorial appendages; so, shield outline; ste_1–5_, sternites. Scales: (*a–c*) 10 mm, (*d*, *e*) 5 mm, (*g*, *i*) 1 mm. Photos: (a), Lilian Cazes.

The digestive system and gills appear dense on the tomograms (white to light grey), indicating preservation in dense material (figure 1*b* and electronic supplementary material, S3*d,f*). The cuticular and muscular structures (e.g. appendages), heart and gonads appear fainter (dark grey to black; figure 1*b*, electronic supplementary material, figure S3*f*,*g*), indicating preservation in a low-density material or as void. Around the posterior segments and appendages, a dense halo is regularly visible (figure 1*b*,*c*). In the central part, a grey area of random shape indicates filling with sediment (figure 1*b*).

These observations are in agreement with a previous study on the preservation of La Voulte thylacocephalans [27]. They indeed found that muscles and cuticle were preserved in fluoroapatite, usually haloed with a sulphide layer. This would explain the low density of these structures and the presence of a dense halo around the posterior segments and appendages in our scans. In the same study, they also noticed that the heart cavity is usually filled with Mg-calcite or dolomite, surrounded by muscles preserved in fluoroapatite, therefore explaining the low density of these structures. Gonad preservation is probably similar to that of the heart, muscles and cuticle. The gills and hepatopancreas are mainly preserved in pyrite, thus explaining their high density and that of the rest of the digestive system.

### (b) Morphological description

#### (i) Shield

Most of the body is covered by a univalve shield (figure 1*a*,*d*). Its univalve nature is demonstrated by the absence of a distinct boundary, such as a hinge line, between both sides of the shield and by the presence of a dorsal fold (electronic supplementary material, figure S1*f*). The shield has a trapezoidal shape in lateral view and is twice as long as high (MNHN.F.R50931: length = 40.0 mm; maximal height = 18.8 mm). The maximal height is reached at about mid-length. The anterior margin forms a large, symmetrical optic notch. The anterodorsal and anteroventral corners are not preserved. The dorsal midline is convex and bears a carina around its mid-length. The posterodorsal and posteroventral corners and posterior margin are not preserved in the segmented specimens. The ventral margin is formed of a straight posterior part bent anteroventrally and a slightly concave anterior part ascending anteroventrally (electronic supplementary material, figure S1*d*).

A large protrusion occurs anteroventrally, just above the narrow, triangular-shaped marginal fold, which gives rise to the inner layer (electronic supplementary material, figure S1*d*,*h–j*). The surface of the shield is entirely covered by horseshoe-like ridges of approximately 190 µm in length, their concavity being oriented anteriorly (electronic supplementary material, figure S1*m*). Three lateral carinae lie on the lateral surface of the shield (electronic supplementary material, figure S1*a,b*): (i) a semi-circular anterior dorsolateral carina, (ii) a posterior dorsolateral carina, which is horizontal posteriorly and becomes vertical anteriorly, and (iii) a straight horizontal mediolateral carina, over almost the entire length of the shield. All carinae are adorned with numerous oval markings (average height of 250 µm and average width of 160 µm). Thirty-one are preserved on the mediolateral carina, 18 on the anterior dorsolateral carina and 29 on the posterior dorsolateral carina.

#### (ii) Tagmatization

The body of *D. ingens* is divided into two broad regions: (i) a cephalothorax encompassing at least the eyes, mandibles, maxillules and maxillae followed by a pair of maxillipeds, three pairs of raptorial appendages and a pair of tubular structures which are most probably appendages, and (ii) a posterior trunk composed of 10 segments, the last 9 bearing homonomous appendages, and a telson.

#### (iii) Cephalothorax

The cephalothorax is formed of at least nine segments. The first one is the ocular segment, which bears a pair of large compound eyes. It is not preserved in the studied specimens here but was extensively described in a previous study [6]. The second preserved segment is only distinguishable by its pair of appendages, no sternite being preserved. They are located just in front of the cardiac stomach and converge ventrally and symmetrically towards the sagittal plane. They consist of a single triangular podomere extended medially. Muscles are attached proximally to this podomere (figures 1*f* and electronic supplementary material, S2*e,h*). These appendages are interpreted as mandibles.

**Figure 2.**
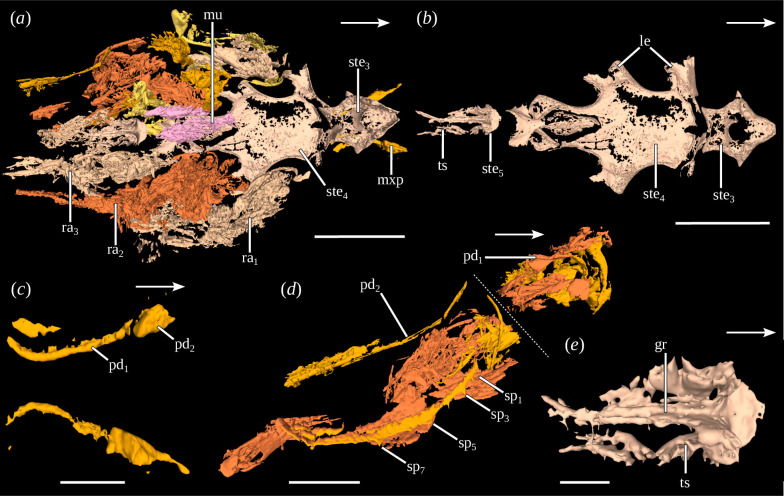
Anterior trunk of *Dollocaris ingens* Van Straelen, 1923 (MNHN.F.R50931). (*a*) Dorsal view of anterior trunk (three-dimensional rendering), (*b*) dorsal view of anterior trunk sternites (three-dimensional rendering), (*c*) dorsal view of maxillipeds (three-dimensional rendering), (*d*) right lateral view of left second raptorial appendage (three-dimensional rendering), (*e*) dorsal view of fifth anterior trunk segment (three-dimensional rendering). White arrows indicate the anterior side. See electronic supplementary material, figure S2, for additional views. gr, groove; mu, muscle; mxp, maxilliped; pd_1,2_, podomeres; ra_1–3_, raptorial appendages; sp_1–7_, spines; ste_3–5_, sternites; ts, tubular structure. Scales: (*a*, *b*) 2.5 mm, (*c–e*) 1 mm.

The third and fourth segments can be distinguished by their sternites, which are preserved under the cardiac stomach (figures 1*e,g* and electronic supplementary material, figure S2*f,g* ). The sternite of the third segment (sternite 1) is poorly preserved: it seems to have a trapezoidal shape, almost square, in dorsal view. It is associated on each side with symmetrical muscular structures, directed posterolaterally. These structures could be remains of appendages (i.e. maxillules; electronic supplementary material, figure S2*d*,*e*,*g*). The fourth segment has a more distinct sternite (sternite 2). It has a trapezoidal shape with both anterior corners expressed as small spines. Paired appendages are inserted laterally to the sternite. They are formed of five podomeres (figures 1*g* and electronic supplementary material, figure S2*d,f,g*). The first two podomeres are cubic and of approximately the same size. The third podomere has a cuboid shape. It is about twice as long as the previous ones. The fourth podomere has a cylindrical shape and is bigger than the others, though it is similar in length to the third one. The fifth podomere is the longest and has a triangular prismatic shape. The joint between the fourth and fifth podomeres is elbow-like, giving the appearance of a subchelae to these appendages, which are interpreted as the maxillae.

The fifth segment has a pentagonal sternite, with the four posteriormost corners expressed as spines (sternite 3; figures 1*e* and electronic supplementary material, figure S2*c,f*). Several muscles, whose morphology and number are undetermined, lie in front of this sternite (figure 1*d*). Below the sternite lies a pair of elongate appendages, anteriorly oriented, which we interpret as maxillipeds (figure 2*a*,*c*, electronic supplementary material, figure S2*j*,*l*). They are formed of at least two podomeres: an elongated and thin proximal one, and a shorter, massive and cubic distal one.

The sixth segment possesses a large sternite on which the three pairs of raptorial appendages are inserted (sternite 4; figure 2*b* and electronic supplementary material, figure S2*i,m*). This segment is probably the result of the fusion of three segments owing to its association with three pairs of appendages (see §4). Lateral extensions occur at the insertion of the raptorial appendages. Despite the cuticle being rarely preserved, the structures and morphology of the raptorial appendages can be studied thanks to the muscles. Only the proximal part of the appendages is preserved. It is formed for each appendage of two podomeres (figure 2*d* and electronic supplementary material, figure S2*k,m*). The first podomere is short, cylindrical in shape, as long as wide and oriented posterodorsally. The second podomere is cylindrical, much longer than wide and posterodorsally oriented. On the second raptorial appendage, this podomere bears four short spines on its anterior side. Additionally, two elongated, paired muscles are preserved dorsally to the sternite (figure 2*a*).

The ninth segment has a thin sternite with a semi-circular anterior part and an elongated posterior part with lateral margins raised into a groove (sternite 5; figure 2*b*,*e*). Two elongated, tubular structures, which are most probably appendages, are protruding posteriorly from the anterior part of the sternite. No elements can be distinguished on it.

#### (iv) Posterior trunk

**Figure 3.**
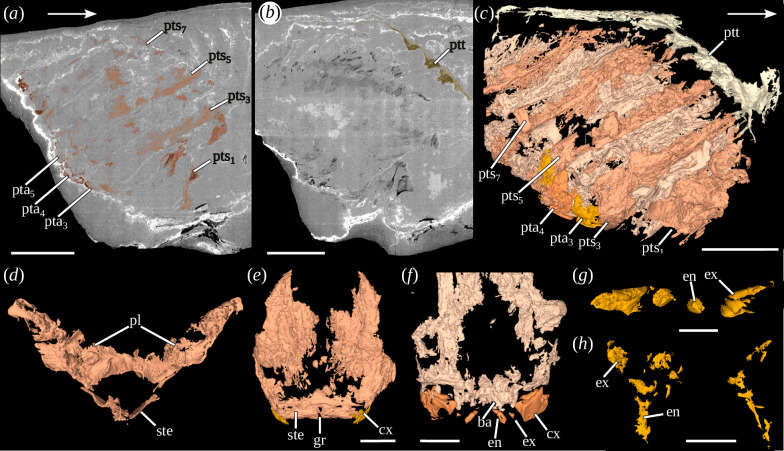
Posterior trunk of *Dollocaris ingens* Van Straelen, 1923. (*a–f*)*,* MNHN.F.R50931. (*a*, *b*) Transverse virtual sections focused on the posterior trunk, (*c*) right lateral view of the posterior trunk (three-dimensional rendering), (*d*) anterior view of the first posterior trunk segment (three-dimensional rendering), (*e*) posterior view of the third posterior trunk segment (three-dimensional rendering), (*f*) posterior view of the posterior trunk segment (three-dimensional rendering), (*g*, *h*) posterior trunk appendages 7 and 4 of MNHN.F.A97320 (three-dimensional rendering), (*g*) dorsal view, (*h*) anterior view. White arrows indicate the anterior side. See electronic supplementary material, figure S3, for additional views. ba, basis; cx, coxa; en, endopod; ex, exopod; gr, groove; pl, pleurite; pta_3–5_, posterior trunk appendages; pts_1–7_, posterior trunk segments; ptt, posterior trunk tergal part; ste, sternite. Scales: (*a–c*) 3 mm, (*f*) 2.5 mm, (*e*) 1.5 mm, (*d*) 2 mm, (*g*, *h*) 500 µm.

The posterior trunk is located in the posterior part of the shield, although it does not appear fused to it, as the tergal part of its segments is well differentiated. It includes 10 segments, mainly discernible by their musculature, and the telson (figure 3, electronic supplementary material, figure S3*b*). Segments are generally much higher than long. The morphology of the first segment is distinct from the others: it is less high than the previous segments and seems more flared in its dorsal part, having a rather trapezoidal morphology (figure 3*d*). The other nine posterior segments are oval to rectangular in shape (figure 3*e*,*f*). Their tergal, sternal and pleural parts are well differentiated (figure 3*b*,*c*). The sternites are crossed in their centre by a groove (electronic supplementary material, figure S3*i*).

The last nine posterior trunk segments are preserved with their appendages. The appendages can be differentiated into four parts (figure 3*f*–*h*): (1) a side plate, associated with the segment pleurites, interpreted as a coxal plate, (2) a central part, originating from the lateral plate, interpreted as the basis, (3–4) two rami branches emanating from the basis, interpreted as the exopod and endopod. The endopods are rather thin and cylindrical, while the exopods are much shorter and appear cubic. The poor preservation of the appendages precludes the observation of podomeres or of annulation.

### (v) Organ systems

Parts of the digestive, respiratory, circulatory and reproductive systems are preserved. The anatomy of the digestive, respiratory and circulatory systems is consistent with previous descriptions [6,28]. A new digestive structure is observed in our specimens: two plate-like structures flank the hindgut in its front (electronic supplementary material, figure S3e). They could correspond to posterior caeca.

Two plate-like structures, flattened dorsoventrally, are also preserved above the hepatopancreas. They are connected to each other in their anterior part, above the cardiac stomach, through an arch-like connective structure. These plate-like structures are interpreted as the gonads (figures 1*d* and 3*c,g,h*). Each of these structures is associated with a long posteroventrally directed canal-like structure, which is interpreted as a genital duct based on its morphology and its connection with the gonads.

A significant number of muscular structures are preserved along the left side of the shield (figures 1*d* and 3*c*). Because of their poor preservation, it is not possible to determine their exact nature and morphology.

## 4. Discussion

### (a) Tagmatization in thylacocephalans

Several hypotheses have been proposed regarding the tagmatization of thylacocephalans (see [29] for a review), all generally involving the definition of two main body regions: an anterior region, comprising the shield and possibly an anterior trunk (or a thorax), and a posterior region which has been usually termed posterior trunk (e.g. [6,30]). However, these hypotheses are distinguished from one another by significant variations in the composition of the body regions or in the differentiation of the trunk. Numerous questions remain about the number, nature and morphology of the various types of appendages, in particular the raptorial ones, which were alternatively considered to be entirely cephalic [6,7], partly cephalic and partly thoracic [3,4,29], or fully thoracic [8,30,31]. The questions of the origin of the shield and whether or not trunk segments were fused to the cephalon to form an anterior region are also particularly important. All these uncertainties have thus led to various hypotheses about the phylogenetic affinities of thylacocephalans.

**Figure 4.**
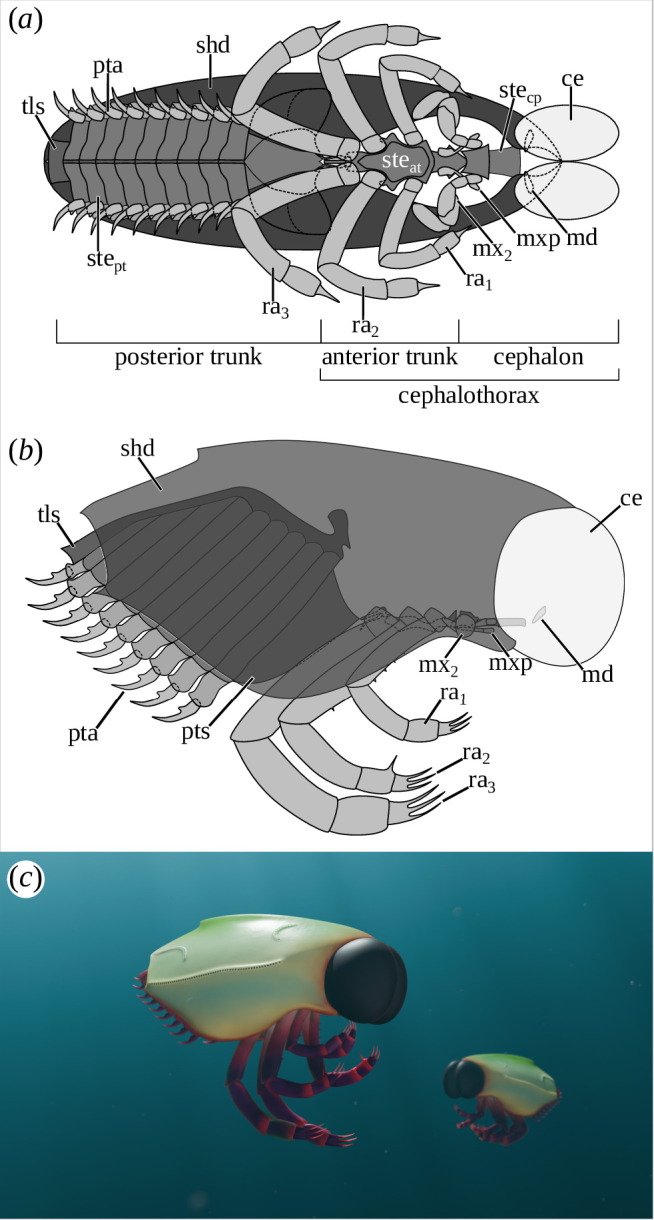
Morphological reconstruction of *Dollocaris ingens* Van Straelen, 1923. (*a*) Ventral view, (*b*) right lateral view, (*c*) artistic reconstruction of two *Dollocaris ingens* in La Voulte-sur-Rhône Basin (reconstruction by Alexandre Lethiers (Sorbonne Université)). Antennula, antenna and maxillae have been omitted from (*a*, *b*) as their morphology is unknown or poorly known. ce, compound eyes; md, mandible; mx_2_, maxillula; mxp, maxilliped; pta, posterior trunk appendage; pts, posterior trunk segments; ra_1–3_, raptorial appendages; shd, shield; ste_at_, sternite of the anterior trunk; ste_cp_, sternite of the cephalon; ste_pt_, sternite of the posterior trunk; tls, telson.

Our results provide major advances on these issues, especially concerning the composition of the anterior region. It was unclear whether it was only formed of the cephalon or if some trunk segments were included in it. Six segments clearly form the cephalon in Thylacocephala: the ocular, antennular, antennar, mandibular, maxillular and maxillar segments (figure 4). The eyes, antennula and antenna are not preserved in our specimens. However, their presence is confirmed in various thylacocephalan taxa (e.g. [3,13,29]). Three additional pairs of cephalic appendages have been discovered here in *D. ingens*. The first of these appendages is formed of a large triangular element, medially enlarged and reminiscent of the gnathal part of the mandible in Mandibulata [32–34]. In addition, its position, next to the cardiac stomach and thus close to the mouth (see [6]), is similar to that of mandibles in pancrustaceans. These appendages are therefore interpreted as such. The presence of mandibles is not surprising as remains of these have been previously reported several times [3,4,30]. However, no palp is preserved in *D. ingens*, contrary to what was reported for *Clausocaris lithographica* (Oppenheim, 1888) [29]. It is also not possible to determine whether or not incisive and molar processes are present on the mandible due to its poor preservation.

A pair of large muscular structures lies posterior to the mandibles. These structures are likely to be appendages (i.e. maxillules), based on their muscular nature, division in two parts, position and orientation, which is similar to the posterior pair of cephalic appendages. This latter has a raptorial morphology that recalls that of the maxilla of Remipedia [35]. By comparison with mandibulates and because of its clear differentiation compared with the appendages of the trunk (see below), this pair of appendages is interpreted as maxilla. Thylacocephala would thus possess a diagnostic mandibulate cephalon with six somites. As no traces of tergites or pleurites have been found, these segment sclerites were likely fused to form the shield, as is often the case in Pancrustacea [36].

Posterior to the cephalic segments, five additional segments have been described in *D. ingens*. As they are clearly differentiated from the cephalic and the posterior regions, we can assume that they are part of an anterior trunk (figure 4). As for cephalic segments, no traces of tergites or pleurites have been discovered for these segments. This observation, as well as the close association of the anterior trunk sternites with those of the cephalic segments, suggests that the anterior trunk segments are part of an anterior region, their tergal and pleural parts most probably being fused to those of the cephalic segments to form the shield. Thylacocephalans therefore had a cephalothorax, i.e. an anterior trunk at least dorsally fused to the cephalon to form a shield [36].

The anterior trunk is composed of at least four, but most probably five pairs of appendages, including the raptorial ones, as previously suggested [8,9,31]. The three raptorial appendages are inserted on the same sternite (sternite 4; figure 2*b*, electronic supplementary material, figure S2*i,m*), which results from the fusion of the individual sternites. The morphology of raptorial appendages also remains debated, more particularly the number of podomeres forming their proximal part. Here, we confirm that the proximal part is formed of two podomeres, the proximal one being short and stout while the distal is long and thin. This is consistent with what has been reported for *C. lithographica* [3].

In front of the raptorial appendages, a previously undescribed trunk appendage has been discovered. Due to its ventral position and forward orientation, it was probably associated with the anterior oral appendages. In several clades of pancrustaceans such as copepods, mystacocarids, remipeds or several malacostracans, one or more thoracic appendages are modified for food manipulation in association with the oral appendages and are thus called maxillipeds [37]. In addition, the elongated and filiform morphology of this newly discovered appendage recalls that of the maxillipeds of other pancrustaceans (e.g. maxilliped 1 of stomatopods; see [38]). It is therefore here interpreted as a maxilliped.

An undescribed pair of tubular structures is located posterior to the raptorial appendages. Their tubular morphology, very different from that of the raptorial appendages and of the posterior trunk appendages, coupled with the absence of annulation and/or podomeres, could suggest that they are not appendages. However, the structures are poorly preserved, making it possible that the elements forming the appendages are not preserved. In addition, the fact that they are paired, symmetrical, tubular structures, extending laterally from a sternite suggests that they might be appendages. These structures are associated with a sternite with a very special morphology (sternite 5), similar to some modified thoracic sternites reported in other pancrustacean ingroups. Some thoracic sternites may be modified in order to form an extension in connection with the reproductive system. This is the case, for example, of stomatopods (seminal receptacle; [39]), copepods (genital atrium and operculus; [40]) or penaeoid prawns [41]. In the last, the sternites of thoracic segments 6–8 are modified to form a large semicircular extension, the thelycum, covering the opening of the spermatheca. It is therefore possible that the fifth anterior trunk sternite in *D. ingens* had a similar function. However, the sex of the specimen is unknown, precluding a direct comparison with the structures mentioned above. Moreover, no direct link is visible with the reproductive system. Indeed, two paired, plate-like structures located in the dorsal part of the studied specimens are interpreted to be the gonads. Similar structures already reported in other thylacocephalans [42,43] were hypothesized as hepatopancreas or gonads. However, a hepatopancreas has already been identified with confidence in *D. ingens* by [6,28], which we also identified in our specimen, just below the putative gonads. The hepatopancreas can be clearly distinguished from the putative gonads by its close association with the midgut, its bilobed or H-morphology and the presence of numerous internal glandular tubes. The paired plate-like structures have on their side a morphology and position more similar to what is known for gonads in other pancrustaceans [44]. It is not possible to assess whether these gonads are ovaries or testicles, due to the lack of other reproductive structures (gonopores, seminal receptacles in females or copulatory structures such as gonopods in males) and the impossibility of studying their ultrastructure. Associated with the gonads is a genital duct. Based on its orientation, the duct could have been connected to the fifth anterior trunk sternite, although no direct link has been observed between these structures in the studied specimens. One could also argue that the large hepatopancreas, which is located just beneath the gonads, would make it complex for the duct to connect to the sternite. Nevertheless, such configurations, in which the duct is curving around the hepatopancreas, are already known from other pancrustaceans [44,45]. This might also be the case for Thylacocephala. More precise data are needed to further characterize the function of this modified sternite.

The presence of a posterior region in Thylacocephala has been known since the 1980s [9,46,47]. However, it was unclear whether this region corresponded to the entire trunk or just to the posterior part of the trunk. With the demonstrated presence of an anterior trunk, we can now confirm that the posterior region is a posterior trunk. The exact number of posterior trunk segments and the morphology of its appendages were also discussed. Eight [6] or 16 segments [2] were previously reported for *D. ingens*. In the studied specimens, 10 segments are clearly preserved in addition to the telson, thus invalidating previous hypotheses. Regarding the appendages, they are here clearly composed of a coxal plate, attached to a stout basis from which two rami emerge, the styliform endopod and a short cubic exopod. These observations confirm the hypothesis of [30] regarding the morphology of the posterior trunk appendages.

A final aspect of the body organization of Thylacocephala has also been the subject of important debates: the origin of gills. Gills have often been thought to be part of an anterior trunk (or thorax; e.g. [6,8,31]). Although well preserved in the studied specimens, the exact origin of the gills cannot be confidently characterized. They do not seem to arise from the posterior trunk, as no connection has been observed with this region. The gills are then probably part of the anterior trunk, as suggested by previous studies. In our specimens, the gills clearly do not arise from appendages. Based on their lateral position, they might originate from the shield. This would be consistent with a previous study made on a Devonian thylacocephalan, *Concavicaris woodfordi* (Cooper 1932), in which the gills appear associated with the inner layer of the shield [43].

To summarize, two tagmata can be distinguished: (i) the cephalothorax, which results from the fusion of the cephalon and the anterior trunk, and (ii) the posterior trunk. No abdomen was found in thylacocephalans, contrary to what was suggested by [7]. This absence of the abdomen, coupled with the differentiation of the trunk into two parts, allows us to definitively reject the affinities with Cirripedia [7]. The division of the trunk also challenges the relationship with Remipedia [3].

### (b) Thylacocephala, the sister group of Malacostraca?

**Figure 5.**
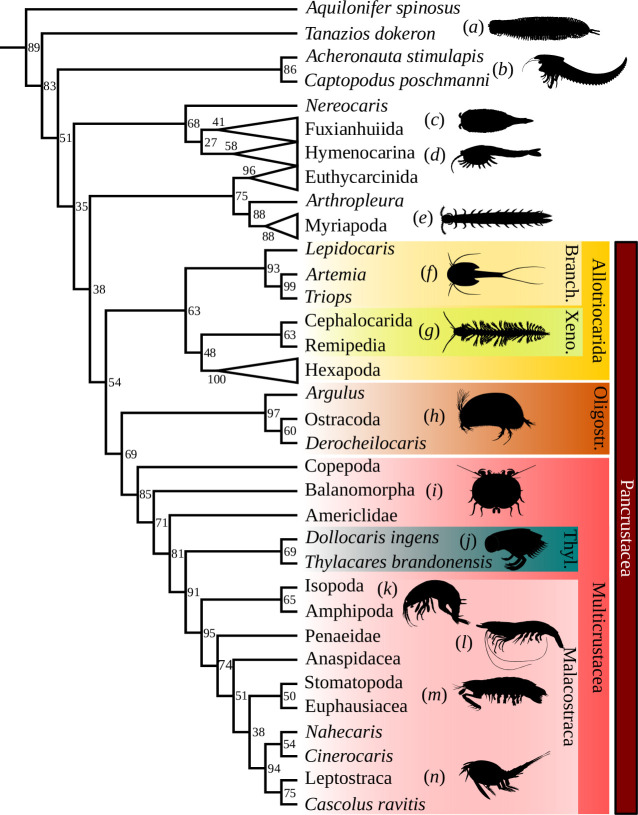
Maximum clade credibility tree of mandibulates (Bayesian inference analysis with constraints). The numbers at the nodes are posterior probabilities (in percentage). See electronic supplementary material, figures S4 and S5 for complete tree and tree from the Bayesian inference analysis without constraints. Branch, Branchiopoda; Oligostr., Oligostraca; Thyl., Thylaco; Xeno., Xenocarida. Except (*J*), silhouettes from Phylopic.org (attributions in electronic supplementary material, document S1).

Phylogenetic analyses recover Thylacocephala as monophyletic with a moderate posterior probability (69% for AC, 66% for AW; figure 5; electronic supplementary material, figures S4, S5). In terms of phylogenetic affinities, thylacocephalan species are placed among Pancrustacea, as already suggested by various authors [3,6,47]. Several morphological character states, such as the presence of an antennule, an antenna and a mandible, already suggested such an affinity. With the discovery of putative maxillules and maxillae in *D. ingens*, the cephalon of Thylacocephala concurs with the structure of the cephalon for Pancrustacea.

Among Pancrustacea, Thylacocephala is resolved as the sister group of Malacostraca in both analyses, with a strong to moderate posterior probability (81 % for AC; 70% for AW). The hypothesis of a relationship to Malacostraca is not new (see [6,8,9]) and has actually already been recovered in a phylogenetic analysis [10]. This is unsurprising as some morphological characters present in thylacocephalans directly relate them to Malacostraca. First, the division of the body with a cephalon, a trunk differentiated into two parts, each bearing appendages, and the absence of an abdomen is well known in Malacostraca, which are the only pancrustacean having a tagmatized trunk, i.e. divided in a thorax (or pereion) and a pleon [48]. In addition, the number of trunk segments is similar between phyllocarids and *D. ingens* (15 in total). This suggests that the tagmatized trunk, with both tagmata bearing appendages, might be a synapomorphy of Thylacocephala and Malacostraca, rather than an autapomorphy of Malacostraca, or that Thylacocephala should be included in Malacostraca. However, the division of segments between both parts of the trunk is quite different (8 + 7 in phyllocarids; 5 + 10 for *D. ingens*). It might suggest that these similar tagmatizations have different origins.

The organization of the digestive system in *D. ingens* is also reminiscent of that of malacostracans. In both groups, the digestive system is divided into: (i) an anterior region, the stomodeum, consisting of the oesophagus and cardiac and pyloric stomachs, (ii) a median region, the mesenteron, comprising the midgut and the dorsal and lateral caeca (e.g. hepatopancreas), and (iii) a posterior region, the proctodeum, formed by the hindgut and rectum [49]. In addition, the newly discovered plate-like paired structure attached to the hindgut of *D. ingens* recalls the posterior caeca, as known in some malacostracans as phyllocarids [50] or decapods [49]. Finally, the presence of maxillipeds is common among eumalacostracans [51], a group that comprises all malacostracans, except phyllocarids. This character state most probably has a strong influence on the position of Thylacocephala as the sister group of malacostracans, but also on the relationships among Malacostraca as shown by our phylogenetic analyses (figure 5). The relationships are indeed quite unusual in our analyses: phyllocarids, which do not bear maxillipeds, are not recovered as the sister group of all other malacostracans (i.e. Eumalacostraca), but are instead nested among eumalacostracans. This could also be explained by the low number of characters focused on pancrustaceans and malacostracans.

## 5. Conclusion

The revision of exceptionally preserved fossils of *D. ingens* from the La Voulte-sur-Rhône Lagerstätte using synchrotron X-ray tomography helped resolve a number of issues related to the anatomy of thylacocephalans. Unique details of its body organization, in particular of its cephalon, have been reported for the first time in our study. We can now confirm the division of the body into: (i) a cephalon, with the presence of mandibles, maxillules, maxillae, (ii) an anterior trunk bearing the raptorial appendages, and (iii) a posterior trunk. These new anatomical data allowed us to test the phylogenetic affinities of thylacocephalans. The phylogenetic analyses strongly suggest the placement of Thylacocephala in Pancrustacea, as the sister group of Malacostraca.

## Data Availability

All original code has been deposited at Zenodo. Original tomographic datasets, triangle-mesh models of specimens (STL format) are publicly available at [52] and [53]. The character matrix and scripts for the phylogenetic analyses are publicly available at [54]. Character lists and changes made to the original matrix are available in electronic supplementary material [55].
